# Recognition of students’ abnormal behaviors in English learning and analysis of psychological stress based on deep learning

**DOI:** 10.3389/fpsyg.2022.1025304

**Published:** 2022-11-22

**Authors:** Mimi Lu, Dai Li, Feng Xu

**Affiliations:** ^1^Common Course Department, Hainan Vocational University of Science and Technology, Haikou, Hainan, China; ^2^School Affairs Office, Hainan Vocational University of Science and Technology, Haikou, Hainan, China; ^3^Department of Information Technology, Guangxi Financial Vocational College, Nanning, China

**Keywords:** psychological characteristics, deep learning, feature recognition, behaviors in English classrooms, students’ behaviors, Python’s Tkinter library

## Abstract

The recognition of students’ learning behavior is an important method to grasp the changes of students’ psychological characteristics, correct students’ good learning behavior, and improve students’ learning efficiency. Therefore, an automatic recognition method of students’ behavior in English classroom based on deep learning model is proposed. The deep learning model is mainly applied to the processing of English classroom video data. The research results show that the video data processing model proposed in this paper has no significant difference between the data obtained from the recognition of students’ positive and negative behaviors and the real statistical data, but the recognition efficiency has been significantly improved. In addition, in order to verify the recognition effect of the deep learning model in the real English classroom environment, the statistical results of 100 recognition result maps are compared with the results of manual marking, and the average recognition accuracy of 100 recognition effect maps is finally obtained, which is 87.33%. It can be concluded that the learning behavior recognition model proposed in this paper has a high accuracy and meets the needs of daily teaching. It further verifies that the developed behavior recognition model can be used to detect students’ behavior in English class, which is very helpful to analyze students’ psychological state and improve learning efficiency.

## Introduction

Psychological stress is a kind of psychological stress reaction produced when people face difficult situations. Research has shown that the right level of stress can motivate people to progress and reach their potential. For students, appropriate pressure can improve their learning efficiency and benefit their growth and development (Wei et al., 2022). However, excessive psychological pressure may bring physical and psychological pain to students, and even lead to the suicidal behavior. If students with abnormal psychological pressure can be found in time, the school can provide timely help and intervention to relieve psychological pressure. At present, psychological stress assessment is mainly conducted through regular questionnaires. However, this method can only reflect the psychological pressure of the students who participated in the survey at that time, and cannot obtain the stress state of the students who did not participate in the survey. In addition, students may fill in the questionnaire perfunctory or intentionally fill in wrong information in order to conceal the real situation (Azitaamirfakhraei et al., 2019). In recent years, the educational data (EDM) mining has made great progress and has received more and more attention. Especially after the direction of machine learning has become hot, the method of machine learning has been integrated into EDM mining, and extracted through deep learning networks. The abnormal behavior characteristics of students in English classrooms can be used to detect and analyze abnormal behaviors such as playing mobile phones and sleeping. A neural network model is established to predict whether students’ academic performance is excellent, good, passed or failed, and various dropout risks.

Classroom teaching activities have always been the focus of research in the field of education. The main body of classroom teaching activities is students. To a certain extent, the students’ classroom behavior status can reflect the classroom efficiency, and it is an important reference index for the classroom quality assessment. With the rapid development of the field of artificial intelligence, school education has gradually become intelligent (Zhou, 2013). Currently, most classrooms are equipped with video recording equipment. These videos record the real behavior status of students in the classroom. For example, by analyzing this data, combining artificial intelligence, deep learning and other related technologies with education, an intelligent behavior analysis system is developed. This has a certain positive effect on the reform of classroom education. The application of deep learning technology is very extensive, and good scientific research results have been achieved in many scientific research fields, especially in image recognition. The field of deep learning is divided into three categories, namely, self-encoding neural networks, deep belief networks, and convolutional neural networks. The most widely used algorithm in deep learning is the convolutional neural network, which has achieved excellent results in the field of image classification. So far, the deep learning technology has played a vital role in promoting industry, transportation, and medical care. However, the research on the combination of computer-related technology and the field of pedagogy is still in the developing stage. Therefore, the classroom behavior research based on deep learning-related technologies is of great value.

According to the theoretical research of pedagogy, there is a certain correlation between the behavior state of students in the classroom and the students’ understanding of the teaching content in the classroom (Tang and Pu, 2014). Therefore, schools can side-evaluate teachers’ classrooms by analyzing the behavioral state results of students in the classroom, which has certain significance for iterative improvement of teaching. The traditional classroom teaching evaluation is generally through the observation of experts, which is not only time-consuming and labor-intensive, but also lacks objectivity (Homer et al., 2018). The development of artificial intelligence, image processing and other fields has brought new solutions to such problems. It is of great significance to apply the deep learning technology to classroom behavior recognition. This can improve the quality of the classroom. Secondly, the convolutional neural network can be applied and developed in various fields. Finally, on the basis of the monitoring system, the automatic detection and recognition of students’ behaviors, and the visualization of the detection results can save teaching resources, so as to achieve the intelligent teaching evaluation. In the future, more and more new technologies will be applied to the information-based teaching environment, which is of great significance to promoting the development of the education field. In addition, students’ behavior in the classroom is also closely related to their psychological state. However, good psychological state is the key to improve learning efficiency. Therefore, the application of in-depth learning technology to classroom behavior recognition can also provide good materials for the judgment and analysis of students’ psychological state, and then help students with abnormal behavior to improve learning efficiency through psychological counseling.

## Related works

With the progress of the times and the development of science and technology, EDM has been widely used. In the traditional teaching environment of face-to-face communication between teachers and students, EDM usually uses statistical methods to analyze the collected data, which is generally obtained through interviews, questionnaires and other methods. However, with the development of information technology, the open teaching environment based on big data education has achieved a great success. EDM begins to apply the data mining technology to the Web environment, such as students’ login logs, homework and teaching resources. At this time, EDM mining methods are mainly divided into three categories: clustering, classification and deviation detection, association rule mining and sequential pattern mining, and text mining. In recent years, with the rapid development of big data and deep learning, deep learning is introduced to solve various common problems in education and teaching, and student behavior and student psychoanalysis is an important research field.

The traditional analysis of students’ behavior in English class is mainly done through teachers’ actual observation. EDM mining, artificial intelligence and other technologies in the research and application of student behavior recognition have only begun to rise in recent years. Lin et al. (2021) proposed to use the images and pose data taken by the classroom camera to identify the students’ behavior. The data processing process is mainly completed through the depth neural network, and the recognition efficiency has been improved to a certain extent after processing (Lin et al., 2021). Wu (2021) using PSO-KNN joint algorithm, we have constructed a student behavior recognition model based on image analysis, with high accuracy. Abdallah et al. (2021) proposed to recognize students’ classroom learning behavior through facial expression recognition and capture, and also to process the identified data set through deep learning method, so as to classify students’ behavior and complete accurate identification of students’ behavior characteristics.

The psychological pressure of college students has been paid more and more attention. Using data mining technology to analyze students’ psychological state has been widely studied. The psychometric evaluation model makes full use of learners’ behavior data, tracks and evaluates learners’ psychological state, and provides reference for students’ psychological adjustment. Effective psychological adjustment can maximize learners’ learning efficiency. Rosa et al. (2019) proposed a knowledge-based recommendation system (KBRS), which includes an emotional health monitoring system to detect potential psychological disorders, especially users with depression and stress. According to the monitoring results, KBRS based on ontology and emotion analysis is activated to send happy, calm, relaxed or incentive information to users with psychological disorders. In addition, the solution also includes a mechanism to send a warning message to authorized personnel when the monitoring system detects a depressive disorder (Rosa et al., 2019).

At present, the commonly used student abnormal behavior detection algorithms include anomaly detection based on data statistics, anomaly detection based on distance, anomaly detection based on density, anomaly detection based on depth and isolated forest algorithm. Machine learning (another important research field of machine, learning) is one of the important research topics in the field of artificial intelligence and neural computing in the artificial intelligence application family born after the emergence of expert systems. And deep learning is an important research field of machine learning, which is the product of its further development.

Based on the current research, in the research, a deep learning model for automatic recognition of students’ behaviors in English classrooms is propose. Students’ English classroom videos are selected as research data and pedagogy-related theories and observation of classroom video laws are combined to define students’ behaviors categories. The main English classroom behaviors include six categories: listening, writing, sitting at the desk, raising hands, standing, and looking left and right. In the research, ResNet50 is selected as the basic classification network. First, pre-training is performed on the public data set. After the network learns the relevant features, it is retrained by parameter transfer to obtain the final model for students’ behaviors classification. For the design and implementation of visualization system, based on the trained model, a visual display system of behavior recognition results is quickly constructed by mainly using Python’s Tkinter library. After the recognition, the psychological pressure of students is analyzed to maximize the value of students’ behavior recognition and provide more help for students’ learning.

## Recognition of students’ English classroom behavior based on deep learning

In recent years, the frequent occurrence of campus safety accidents has attracted great attention from all walks of life. In the construction of smart campus, the abnormal behavior prediction of students in campus safety plays a very important role. Therefore, an intelligent prediction system for abnormal behavior of students in English learning based on deep learning is proposed. Deep learning is a branch of machine learning. Many traditional machine learning algorithms have limited learning capabilities. The increase in the amount of data cannot continue to increase the total amount of knowledge learned. Deep learning systems can improve performance by accessing more data and discovering experience (Dai et al., 2019). Learning is carried out by discovering intricate structures in empirical data. In the research, by collecting students’ English classroom videos as research data, combining pedagogy-related theories and observing classroom video rules, students’ behavior categories are defined, deep learning algorithm is used to build a students’ behaviors prediction model, and a complete parallelization of student anomalies is developed. A complete system for predicting abnormal students’ behaviors with parallelization capability is developed.

### Constructing a database of students’ English classroom behavior

Data set is an important basis for designing and validating algorithms, and a high-quality data set has an important impact on the training effect of network models. In order to obtain a behavior recognition model with better performance, the research needs a high-quality data set. However, because the research in this field at home and abroad is still in the development stage, there is no public database on students’ behaviors for scholars to study. Based on this, the initial work of this research is to construct a real English classroom students’ behaviors data set.

#### Image preprocessing

In this experiment,18 teaching videos of different English classes in a university in Haikou in the second semester of 2020 are collected, each with about 40 min, as the original experimental data (the collected teaching videos are used in this research, and the students, teachers and other participants’ consent have been obtained). The data processing process is as follows.

The first step is data filtering and processing. The non-English classroom scene parts in the video are removed, and Open CV is used to uniformly sample the English classroom video to generate a picture dataset in jpg format. After the first step of processing, a total of 72,440 pictures of students’ English classroom pictures are collected and sorted (Manju and Valarmathie, 2021).

The second step is image noise reduction. Due to the complexity of the English classroom environment, the collected frame images inevitably contain noise, which will inevitably affect the final recognition result. Therefore, some methods such as mean filtering, median filtering, bilateral filtering and Gaussian filtering are used in the image preprocessing process. It is very necessary to denoise the image data with algorithms.

**Figure 1:**
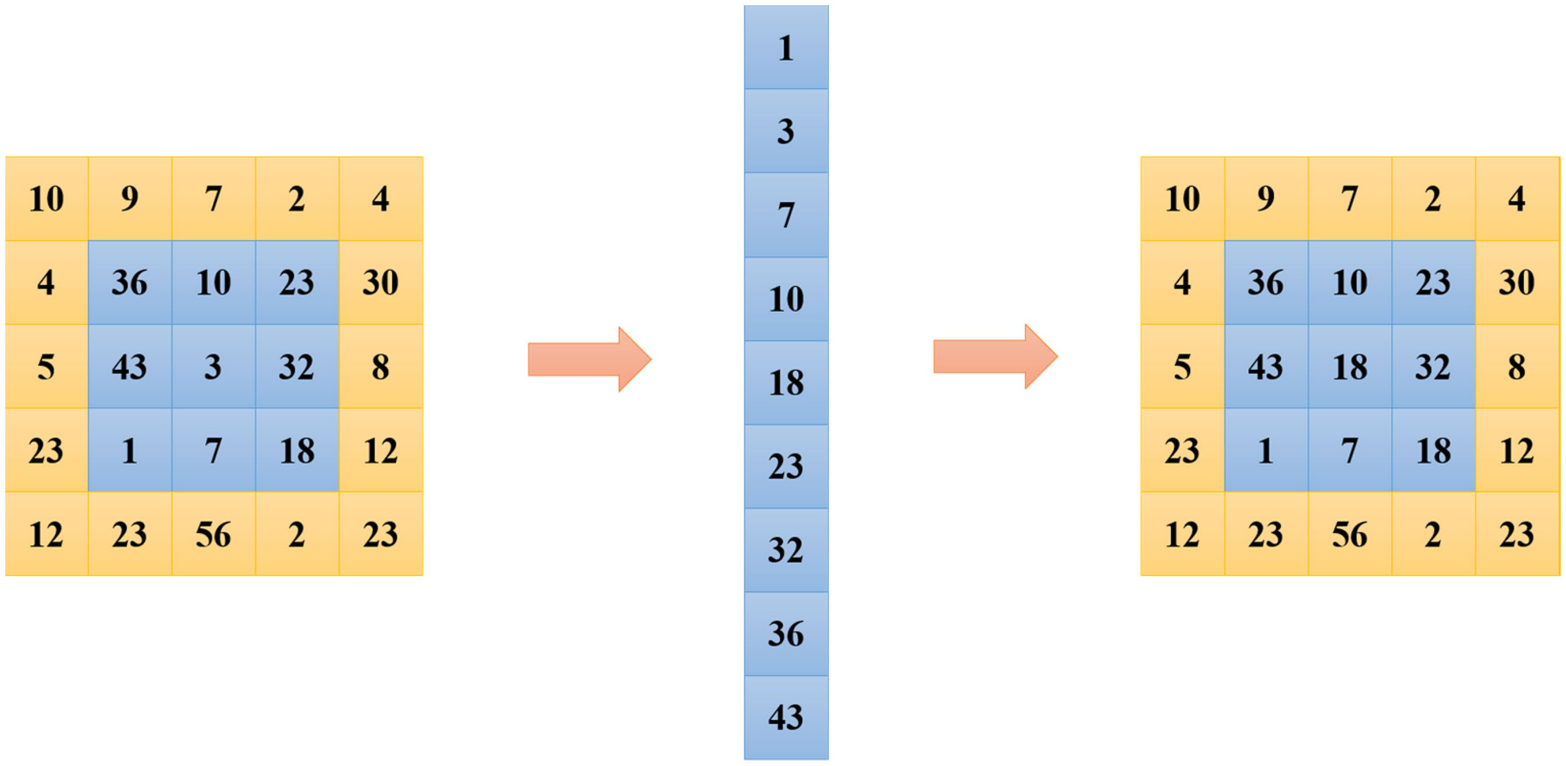
Median filtering.

(1) Median filtering is a nonlinear smoothing algorithm. As shown in Figure 1, a matrix in the shaded part of the figure is taken as a sliding template, and the pixel values are sorted and then the median value is obtained. Such an operation facilitates the elimination of isolated noise points. In the specific operation process, several sliding templates can be selected to slide on the image matrix, such as diamond, rectangle, circle, etc. The median filter formula is shown in Formula (1).


(1) g(x,y)=med{f(x−k,y−l),(k,l∈W)}

In Formula (1), W is a two-dimensional template. This method can protect the edge information of the image well and remove the salt and pepper noise.

**Figure 2:**
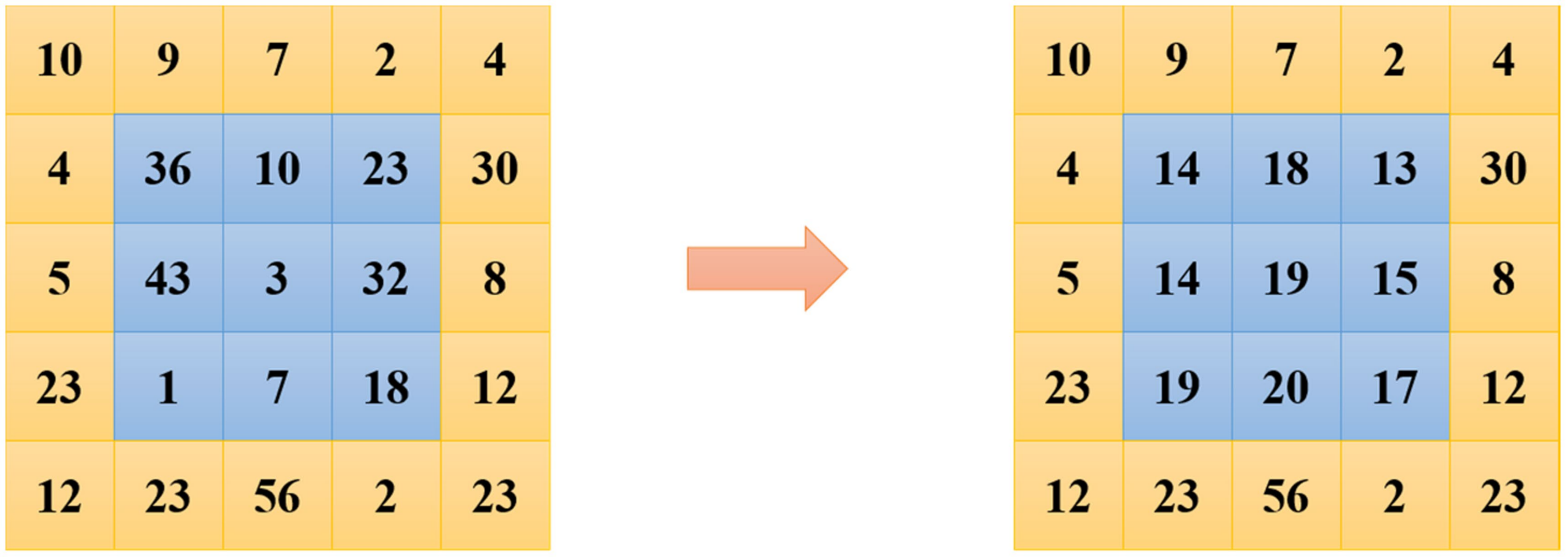
Mean filtering.

(2) The most important method of mean filtering is the neighborhood averaging method. Each pixel in the image takes the average value of its neighborhood, as shown in Figure 2, and the calculation formula of the algorithm is shown in Figure 2.


(2) $$f\left(m,n\right)=\frac{1}{9}\overset{1}{\underset{i=-1}{\sum }}\overset{1}{\underset{j=-1}{\sum }}f\left(m+i,n+j\right)$$

Although the algorithm is simple and has a fast rate, the disadvantage is that it cannot completely retain the detailed information of the image, especially the edge information of the image is lost, so it cannot remove noise points well.

Gaussian filtering can be understood as a smoothing algorithm for data. As long as it is noise that obeys a normal distribution, Gaussian filtering can play a good inhibitory role on it. The Gaussian filtering algorithm process is actually a weighted average operation process on the entire image. In detail, it is to first use a template to perform a smoothing operation on the image, and then perform a weighted average calculation on all pixels in the template domain. The result is used as the center pixel of the template. Formula (3) is the core of Gaussian filtering (Bhattacharjee and Das, 2020).


(3) $$G\left(x,y\right)=\frac{1}{2\pi \sigma^{2}}e^{-\frac{{\left(x-a\right)}^{2}+\left(y-b\right)}{2\sigma^{2}}}$$

The difference from the mean filter is that the Gaussian filter algorithm increases the distance condition, and the weight will gradually decrease as the distance between the pixel point and the center pixel point increases. Gaussian filtering can preserve all the details of the image to a greater extent and reduce the phenomenon of information loss.

(4) The bilateral filtering algorithm is a nonlinear filtering algorithm. The weighted average calculation method it uses is very similar to other filtering algorithms. The pixel value is represented by the weighted average value obtained from the neighborhood pixel value. The spatial domain and the pixel range domain are two important concepts of the bilateral filtering weight domain. The weight of the spatial domain is expressed as *G_s_*, and its calculation formula is:


(4) $$G_{s}=\exp\left(-\frac{{\left\|p-q\right\|}^{2}}{2\sigma_{s}^{2}}\right)$$

Suppose *q* represents the center of the window, *p* to be a certain point, and $G_{r}$ represents the weight of the pixel range, and its calculation formula is:


(5) $$G_{r}=\exp\left(-\frac{{\left\|I_{p}-I_{q}\right\|}^{2}}{2\sigma_{r}^{2}}\right)$$

The filtering result of bilateral filtering can be expressed as:


(6) $$BF=\frac{1}{W_{q}}\underset{p\in s}{\sum }G_{s}\left(p\right)G_{r}\left(p\right)\times I_{p}$$

In Formula (6), *W*_q_ represents the sum of the weights obtained by adding the weights of each pixel in the corresponding filtering window. This function protects the edge information of the image by acting differently on the flat area and edge area of the image.

#### Definition of students’ behaviors

**Figure 3:**
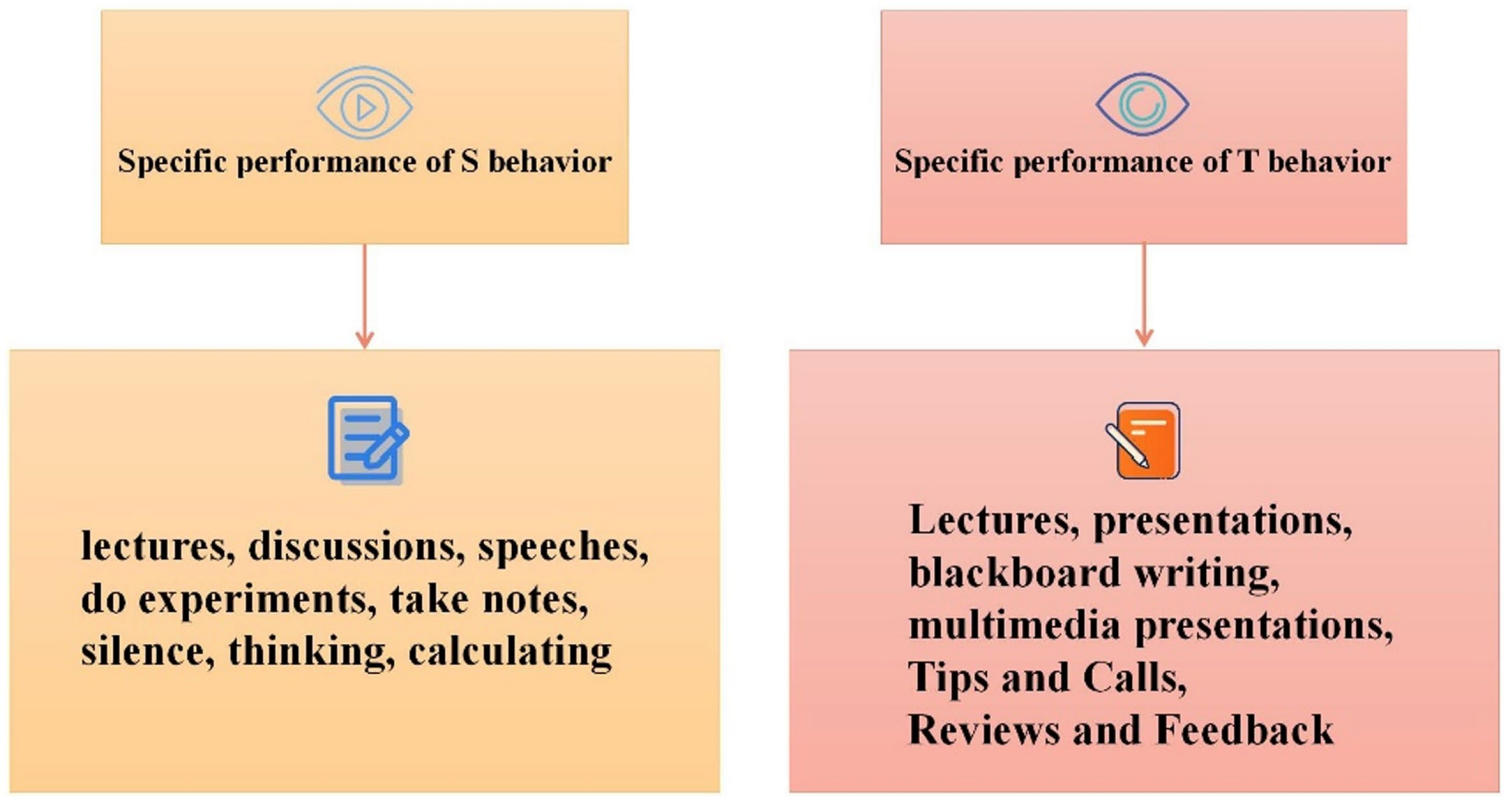
S-T behavior specific performance.

In English classrooms, students’ behaviors are generally complex and changeable. From different perspectives, it can be divided into different categories. For example, from the perspective of English classroom, it can be divided into active classroom and passive classroom. From the perspective of the main body of the classroom, that is, students, it can be divided into the learning state and the non-learning state. According to the statistics of education related surveys, only 43% of the students will ask questions in English class, 57% of the students will not ask questions, among them, 17% of the students will not ask questions (Lin et al., 2021). Students are afraid to ask questions, and 40% of students prefer to ask their teachers after class to answer questions. In the English learning class, for example, whether students actively ask questions can not only reflect the activity of the English learning class, but also reflect the students’ mastery of the knowledge taught by the teacher in the English learning class to a certain extent. Analysis is helpful for teachers to grasp the situation of students and evaluate the English learning classroom. The students’ behaviors selected in the research combines the S-T (Student-Teacher) behavior analysis method in the pedagogical theory. Figure 3 shows the specific performance of the teacher and students’ behaviors in the English learning classroom.

Finally, several common behaviors of students in English learning classroom videos are counted in the research. According to the S-T behavior analysis method, the behaviors of students with relatively high frequency in the videos listed in Table 1 below are selected for research. The detailed discriminant criteria for these behaviors are shown in the table.

**Table 1:** Discrimination of students’ behaviors in English learning classroom.

Behavior name	Discrimination standard
Stand	Students are upright
Raise one’s hand	Hold up with one hand
listen to a talk	Sit more upright, look up to the blackboard direction
Write	The student had a pen in his hand and kept his head down
Look right and left	Student’s body turns left and right, or behind, the facial visibility rate is small
Lie on the table	The student lay with his head on the table

#### Student location detection based on Yo lo_v3

Training the students’ behaviors classification model requires a large amount of student image data, but using an annotation tool such as label I mg to manually label the workload is too much. Therefore, the research adopts the method of target detection to identify the location of the target student, and then according to the returned location information crop the image with Open CV, which not only reduces a lot of repetitive and tedious work, but also obtains student image data with as little irrelevant background as possible (Yu, 2020). At present, the widely used target detection methods include Faster-CNN and methods based on the Yo lo framework. The main feature of Faster-CNN is high accuracy, but the real-time detection effect is not good, and Yo lo uses a fully automatic end-to-end. The end-to-end method to achieve target detection not only has high accuracy, but also ensures the real-time detection. So, in the research, Yo lo_v3 is used to detect student objects in images (Lin and Lai, 2021).

#### Statistics of students’ behaviors data

So far, through the previous algorithm, a basic students’ behaviors database has been obtained, and the description of the relevant attributes of the database is shown in Table 2.

**Table 2:** Description of the attributes of the students’ behaviors data set.

Database properties	Price
The number of pictures	72,440
Multiple grades	√
Multiple collection periods	√
Multiple disciplines	√

**Figure 4:**
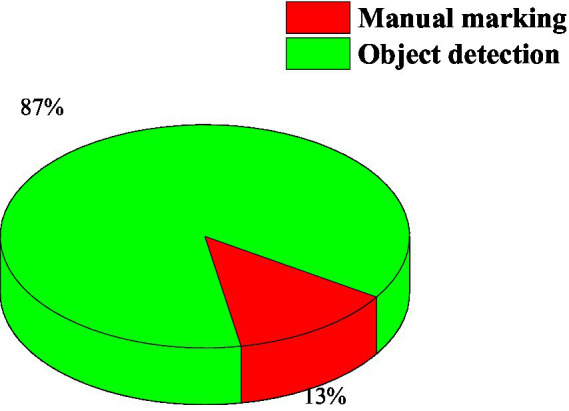
The image situation obtained by the target detection and the manual annotation.

Since the Yo lo_v3 algorithm cannot guarantee that all the student targets in the picture are detected, for the targets that cannot be detected, the labeling tool label l mg is used for manual labeling. After the final statistics, among the 72,440 student images of the total number of behaviors, the total number of manually labeled images is 9,706. Figure 4 shows the proportion of student images obtained by the two methods of Yo lo_v3 detection and manual labeling. It can be found that the Yo lo_v3 algorithm can basically detect most of the student targets, saving most of the time required for manual annotation. As shown in Figure 4.

**Figure 5:**
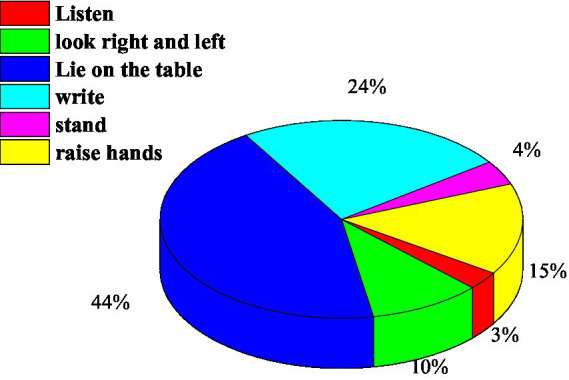
Image number distribution of six categories of behaviors.

Finally, the number of student images of various behaviors is counted, the situation is shown in Table 3, and the distribution ratio is shown in Figure 5.

**Table 3:** The amount of data for each behavior category.

Action	Listen to a talk	raise one’s hand	Stand	Write	Lie on the table	Look right and left
Number	31,877	10,888	2,896	17,389	2,178	7,233

#### Data enhancement of students’ behaviors

From the data display analysis in the previous section, it can be seen that the distribution of image data of students in various categories is not balanced, especially the proportion of data in the categories of “lying on the desk” and “standing” is relatively small. After that, the classification effect of the classification model needs to further expand the data set. Considering that it is difficult to collect new data to expand the data set, the research uses data augmentation methods to obtain more data through geometric transformations, such as rotation, flip, or color transformation. The principle of data enhancement is to obtain a new image by changing the spatial position relationship of pixels in the image. And coordinate transformation and grayscale interpolation are the two main operations of spatial transformation. Formula (7) is the spatial coordinate transformation formula.


(7) (x,y)=T{(v,w)}

In Formula (7), (v, w) is the coordinates of the pixels in the original image, and (x, y) is the pixel coordinates of the transformed image. Formula (8) is the spatial coordinate transformation of affine transformation, which is one of the most commonly used spatial coordinate transformations.


(8) $$\left[xy1\right]=\left[vw1\right]T=\left[vw1\right]\left[\begin{array}{l} t_{11}t_{12}0\\ t_{21}t_{22}0\\ t_{31}y_{32}1 \end{array}\right]$$

Table 4 illustrates the matrix values used to implement these changes.

**Table 4:** Principle of affine transformation algorithm.

Change the name	The affine matrix T	Coordinate formula
Identity transform	1 0 0	x = v y = w
	0 1 0	
	0 0 1	
Scale transformation	c_x_ 0 0	x = c_x_v y = c_y_w
	0 c_y_ 0	
	0 0 1	
Rotation transformation	cosθ sinθ 0	x = vcosθ-wsinθ y = vsinθ+wcosθ
	- sinθ cosθ 0	
	0 0 1	
Translation transformation	1 0 0	x = vsv + w y = w
	0 1 0	
	t_x_ t_y_ 1	

In the research, geometric transformation and color transformation are mainly used for data enhancement. The specific methods are as follows.

1. Translation transformation: move the image content along the horizontal or vertical axis;
2. Flip change: flip the image along the horizontal or vertical direction;
3. Rotation changes: mainly left and right rotation, random rotation;
4. Some pixel values are randomly set to zero;
5. Mean fuzzy and median fuzzy processing;
6. Contrast transformation: that is, changing the brightness of the image;
7. Noise disturbance: disturb the image by using some noise;
8. Color change: make appropriate adjustments to the extracted R, G, B component values;

After the data augmentation operation, the data volume of various behaviors is shown in Table 5. The data expansion is mainly carried out on the students’ behaviors categories with a small amount of data originally. After expansion, the total amount of students’ behaviors image data increased from 72,440 to 90,987.

**Table 5:** The amount of image data of each behavior category after the data enhancement.

Action	Listen to a talk	Raise one’s hand	Stand	Write	Lie on the table	Look right and left
Number	31,877	10,888	10,299	17,389	10,276	10,258

### The algorithm flow of students’ English learning classroom behavior recognition

**Figure 6:**
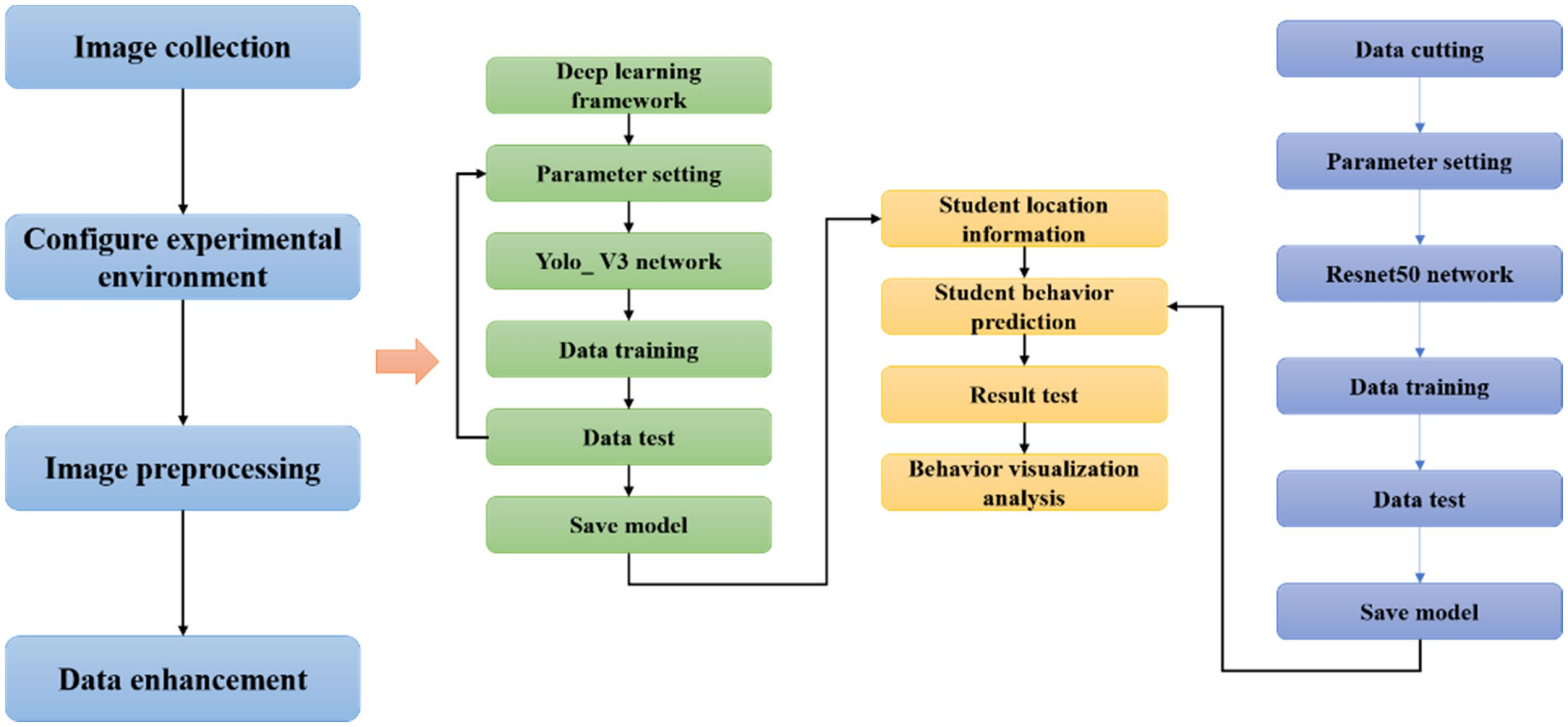
Algorithm flow chart.

In the research, the transfer learning method based on ResNet50 network is mainly used to realize the detection and recognition of students’ behaviors in English learning classroom. The whole research process is shown in Figure 6.

Step 1: Collect student image data and configure the experimental environment.

Step 2: Preprocess the collected data. Firstly, the position information of the students in the picture data is obtained through the target detection algorithm, and then the irrelevant background is cut out according to the position information and only the target area is retained, and then several common data enhancement methods are used to expand the training set.

Step 3: Train the network. The ResNet50 network pre-trained on the ImageNet public data set is used as the behavior classification model, and then the self-made students’ behaviors data set is used for training. The final classification model is used for later behavior recognition of student images.

Step 4: Analyze the recognition results of students’ English learning behaviors. Tkinter in the Python library is used to simply build an interactive tool to visualize the results, which is convenient for subsequent analysis and evaluation.

## Experimental verification and analysis

Through the recognition and analysis of students’ behaviors, the overall class status of students can be obtained to a certain extent, and a new method for classroom evaluation of English learning can be provided. The traditional human behavior recognition is based on the method of hand-designed features, and later a classification algorithm based on deep learning to automatically extract features is implemented. At present, the research on human behavior mainly focuses on the large action scenes of outdoor sports, but there is still a lack of research on the detailed actions of students’ learning behaviors in English learning classrooms. The state of students’ learning behavior in English learning classroom is, to a certain extent, the feedback of teachers’ teaching effect. Therefore, this research identifies students’ behaviors, and then visualizes and analyzes the data of the identified results. This is of great significance for forming an effective teaching orientation and promoting the improvement of teaching quality. In order to verify its feasibility and practicability, the deep learning-based student English classroom behavior recognition method described in the research is applied to a real classroom environment.

This paper uses 10 fold cross validation method to train and test the model. This method is widely used in the field of deep learning, especially in small data sets, so that each sample can be used as both a training set and a test set, and also make it more suitable for our sample set. When dividing training and test sets, take 1/10 of the size of the class data from each group in order as the testing data, and the rest as the training data. Repeat the operation for 10 times to build 10 training sets and test sets with different data. Then train the model on 10 training sets respectively, test the model performance on the corresponding test set, and finally take the average of the 10 test performance as the final performance of the model. The data results recorded in the actual experiment are the average value after 10 fold cross validation.

### Experimental subjects

The original data of the teaching video used in this verification experiment comes from the intelligent classroom. There are two cameras above the classroom, the camera in the middle records the teacher’s behavior video on the podium, while the camera in the front of the classroom is used to record the learning status of students in the classroom (Zhang et al., 2019). Because students’ behaviors are studied, the video recorded by the camera at the front of the classroom is used as the source of the students’ behaviors data set. The participants of this experiment are 30 students in H city and their English teachers (the teaching video collected as the data set of this study has been approved by the school, students, teachers and other participants).

### Experimental method

**Figure 7:**
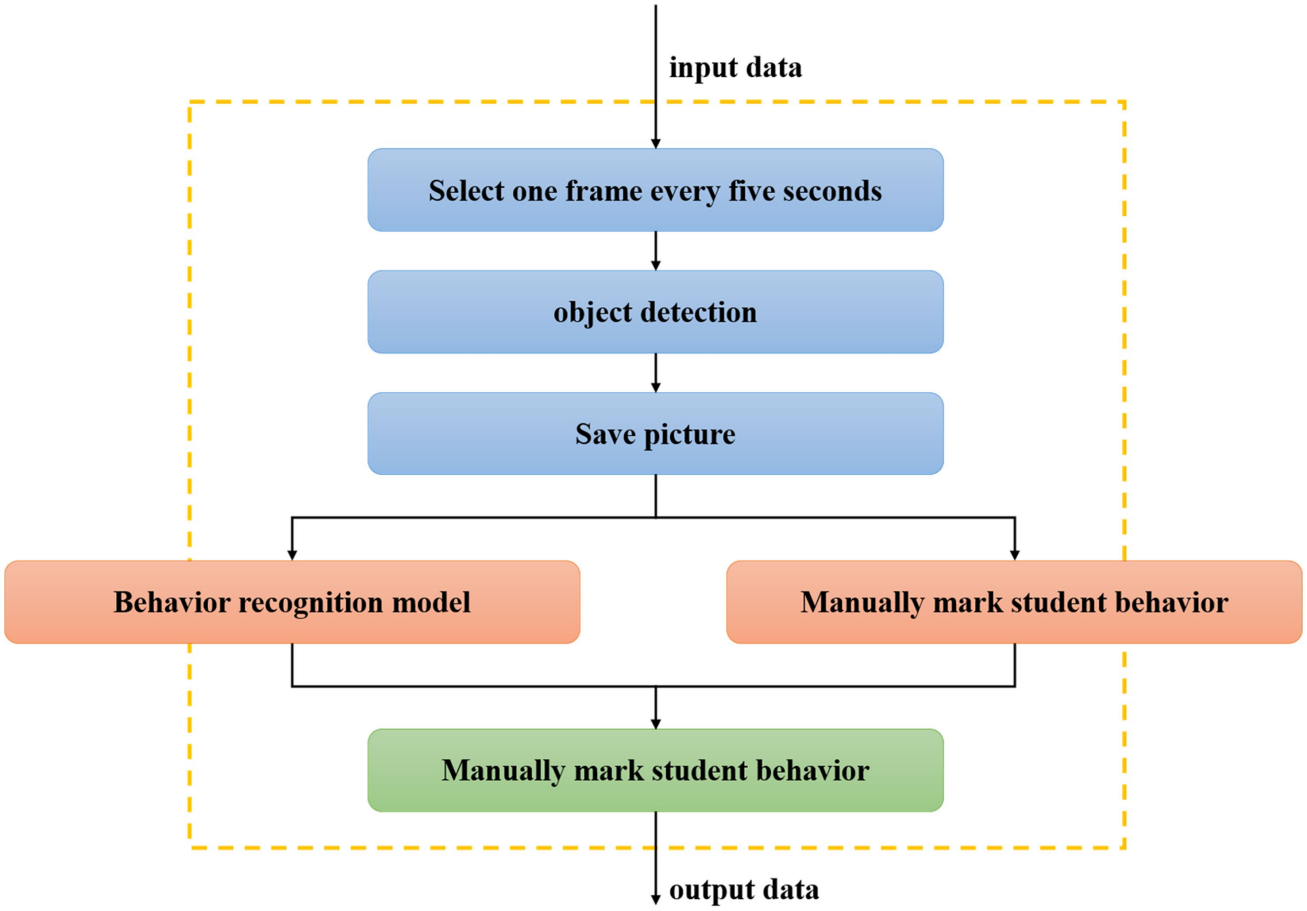
The flow chart of the validation experiment.

The research intercepts a 500-s English learning classroom video as the test data. This video is a clip of teachers explaining English learning in a new class. One frame is selected as a test image every 5 s, and then the trained students’ behaviors recognition model is used to recognize the behavior of the students in the image according to the time sequence of the extracted frame images, and the recognition result graph is obtained (Pang, 2021). In addition, by observing students’ behaviors in a large number of classroom videos and combining with relevant pedagogical theories, the research divides students’ learning behaviors into two categories: positive learning behaviors and negative learning behaviors, as shown in Table 6. While the machine recognizes the student’s behavior, the saved image is manually recognized and the result is recorded. The process is shown in Figure 7. Then the data is counted, and the results of manual recognition and machine detection are compared to verify the accuracy of the experiment. Finally, the result data of the experiment is visualized.

**Table 6:** Classification of students’ learning behaviors.

Serial number	Study behavior classification	Corresponding action
1	Positive act	Write, listen, stand, raise your hand
2	Negative act	Lie on the table and look around

### Verification methods and standards

In order to ensure the accuracy and objectivity of the verification method in the research, it is very necessary to formulate the standard of manual labeling before manual labeling, so the labeling standards shown in Tables 7, 8 below are formulated. In addition, in order to prevent a certain degree of subjectivity from marking by one researcher, the manual marking work in the research is carried out by two researchers at the same time. According to each video frame image, the total number of students and the number of students in each behavior category are recorded. And the total number of positive behaviors, negative behaviors and the ratio of positive behaviors are calculated, and the record form is filled in while marking. Finally, the recorded results of the two researchers are compared, and the number of consistent observations is counted, and then the percentage of the total number of consistent observations is calculated. As a result, the coincidence percentage of the digital records obtained by the two researchers is 90.11%. It can be seen that the manual marking results of the research have a certain degree of objectivity (Ye et al., 2021).

**Table 7:** The standards of the manual marking.

Name (of a thing or organization)	Criterion for judgment
Number of students’ goals	When the student’s shielding area is less than 30%, it is judged that there is a student target here
Positive behavior rate	The ratio of class positive behavior is the number of positive behaviors in the whole class divided by the total target number
Student behavior	Target student behaviors were classified into positive and negative behaviors

**Table 8:** Discrimination of students’ behaviors in English learning classroom.

Behavior name	Discrimination standard
Stand	Students are upright
Raise one’s hand	Hold up with one hand
Listen to a talk	Sit more upright, look up to the blackboard direction
Write	The student had a pen in his hand and kept his head down
Look right and left	Student’s body turns left and right, or behind, the facial visibility rate is small
Lie on the table	The student lay with his head on the table

Finally, after the 500-s test video is detected by the network model, the obtained detection result images are manually counted, and the results are compared with the manually labeled results to verify the accuracy and feasibility of the deep learning model.

### Experimental verification and analysis

The English learning classroom video used in the research is in MP4 format, with a size of 1920*1080, a duration of 500 s, and a total of 100 video images are captured every 5 s. Two researchers calculated the number of students, the ratio of students’ behaviors and positive behavior to the 100 images according to the criteria in Tables 7, 8 above. The following Table 9 shows the recorded results of the number of students, the number of positive behaviors, and the number of negative behaviors selected by manual recognition and machine recognition every 50 s.

**Table 9:** The condition of students’ behavior in the classroom.

		0	50	100	150	200	250	300	350	400	450	500
Artificial perception	Number of students	30	29	27	25	11	23	24	25	25	26	27
	Positive act	23	21	23	22	6	14	17	20	19	20	19
	Message behavior	7	8	4	3	5	9	7	5	6	6	8
Machine cognition	Number of students	29	26	23	21	23	15	17	18	18	21	21
	Positive act	22	20	20	17	21	8	12	14	13	14	12
	Message behavior	7	6	3	4	2	7	5	4	5	7	9

It can be seen from the table that the positive and negative behaviors of students identified by the machine are not much different from the real data. In order to verify the recognition effect of the deep learning model in the real English learning classroom environment, the statistical results of the 100 recognition result maps are compared with the results of manual marking, and the number of correct students’ behaviors detected in each image is calculated and divided by the total recognition in this image. The number of behaviors, that is, the recognition accuracy rate of a single image, and finally the average recognition accuracy rate of 100 recognition result maps is taken, and the result is 87.33%. It can be concluded that the behavior recognition model has an accuracy of 87.33% for students’ behaviors recognition in the real English learning classroom environment (Zhao, 2021).

**Figure 8:**
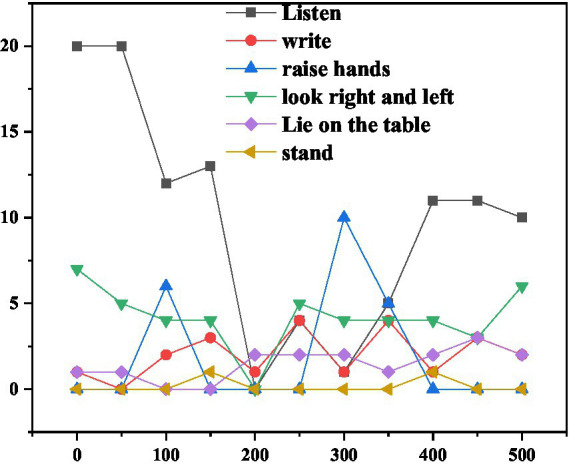
Statistics of the number of behaviors in the class.

A machine recognition result map is selected every 50 s, and the visualization results shown in Figure 8 can be obtained after statistics related to each behavior category.

**Figure 9:**
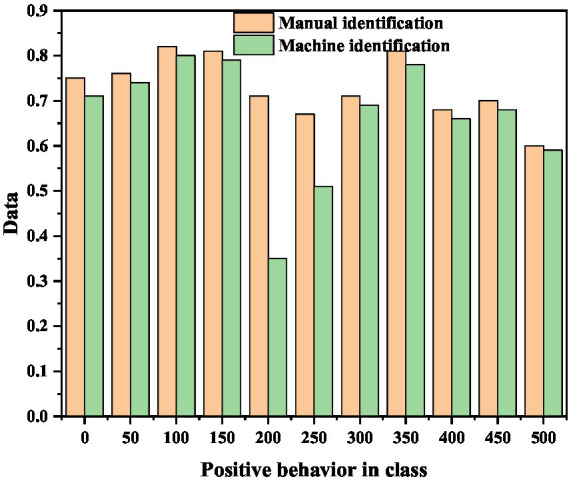
The trend chart of positive behavior ratio change in class.

It can be seen intuitively from the figure that on the whole, the number of students in the “listening” category has slightly decreased over time, which is consistent with the fact that students’ attention in the real English learning classroom will gradually spread as the class time progresses. At 300 s, the number of people who raise their hands is the largest. Combining the video and video, it is found that at this moment, the teacher is checking the small exercises in the English learning classroom completed by the students who raise their hands. A standing classmate is detected at the 150th and 400th seconds, respectively. Combined with the video, it is found that there is indeed a classmate answering the teacher’s question at each of these two moments. According to the recorded experimental data, the positive behavior ratio of the class is selected every 50 s for analysis. The results are shown in Figure 9 below (Zhang, 2022).

As can be seen from the figure, the positive behavior ratios of machine recognition and manual recognition are on the whole gradually decreasing. The trend is subdivided into three stages: positive rising, then falling, and then rising again. At the beginning, the overall enthusiasm is low. Combining the video and audio recording, it is found that the students just returned to the classroom after the morning exercise and did not feel like learning. From 0 to 150 s, the overall enthusiasm of the students has increased. During this period, the teacher talks about the new curriculum, and the learning of new knowledge attracts the attention of the students. Thus, the rate of positive behaviors in English learning classrooms is on the rise. There is a huge difference between the positive behavior rate of machine recognition and the positive behavior rate of manual recognition at 200 s. From the analysis of the English learning classroom video, it is found that at 200 s, most students looked down at the textbook, causing serious occlusion by the students behind and failure of behavior detection. There has been a massive decline in machine-recognized positive behavior.

From the general trend of students’ learning behaviors in English learning classroom, some rules can be obtained. For example, the students’ positive behavior ratio at the beginning of the class is low, and it is difficult for students to quickly enter the English learning classroom state. Teachers can design interesting English learning classroom activities to attract students’ attention and make them quickly enter the learning mode. Through the video analysis, it can be seen that teachers’ teaching methods also have a great impact on students’ learning behavior. Future research can further explore the advantages of different teaching methods by analyzing the trend of students’ behavior changes under a variety of teaching methods. Inferior. The traditional English learning classroom teaching evaluation method is often subjectively scored by multiple subjects such as students, teachers themselves, peer teachers and college leaders according to a series of evaluation indicators and then weighted. Due to strong subjectivity and many human factors, this evaluation method has great narrowness and limitations. This research is to overcome the defect of multi-subject subjective evaluation. From the perspective of analyzing students’ behavior in English learning classroom, it provides a more intelligent method for evaluating students’ classroom participation and enthusiasm (Motevalli et al., 2020).

### Analysis of students’ classroom psychological stress

It can be seen from the classroom behavior recognition that the students’ psychological state, such as the appearance of textbooks blocking their faces, lying on the table and other behaviors, can show the students’ negative emotions. When negative emotions occur, there may be psychological pressure in the classroom, the classroom psychological pressure refers to a classroom psychological state that affects the teaching efficiency, which is formed in the course of classroom teaching based on the interaction between teaching subjects. As an intermediary factor between learning psychological activities and learning personality characteristics, through students’ learning motivation, autonomous learning, learning emotional experience and evaluation effect, the classroom psychological pressure restricts the effect of students’ classroom learning. That is to say, under different classroom psychological pressures, due to the great differences in students’ learning enthusiasm, learning interest, learning emotional experience, and learning attention, the intellectual activities and operational activities in learning are also quite different, resulting in a big difference in students’ learning effects. Under the positive and healthy psychological pressure, students have an open mind, quick thinking, rich and active imagination, enhanced memory, strong energy, active learning, and can better accept new knowledge, and analyze, synthesize, and analyze new knowledge on the basis of new knowledge. The negative and suppressed classroom psychological pressure makes students’ thinking narrow, their intellectual activities are suppressed, and their attention is distracted. Although students are forced to accept certain knowledge, it is difficult for them to think independently, let alone actively explore new knowledge, which is not conducive to their creative learning (Guay, 2022).

## Conclusion

In English learning classroom teaching, teachers will obtain teaching feedback by observing students’ facial emotions or behavioral states in class. However, in the classroom, the behavioral state of the students is changeable and complex, and the teacher’s main focus is on teaching, and it is difficult to fully understand the situation of the students in the classroom. In recent years, with the rapid development and wide application of emerging technologies such as big data and artificial intelligence, artificial intelligence and other technologies have driven the reform of traditional teaching concepts, models, content and methods, and contributed to the informatization and intelligence of education. The research aims to investigate the classification, evaluation and classroom participation analysis of students’ behaviors in English learning classroom through a deep learning-based target detection and students’ behaviors recognition technology, and design and develop an analysis tool for students’ classroom behavior recognition results. It is convenient for the visual analysis and evaluation of students’ classroom behavior and enthusiasm in the teaching process. From the overall trend of students’ classroom learning behaviors, some rules can be obtained. For example, the score of positive behavior ratio of students at the beginning of class is low, so it is difficult for them to quickly enter the state of English learning class. Teachers can design interesting English learning classroom activities to attract students’ attention and make them quickly enter the learning mode. By combining the video analysis, it can be seen that teachers’ teaching methods also have a great impact on students’ learning behaviors. Future research can further explore the advantages and disadvantages of different teaching methods by analyzing students’ behavior change trends under various teaching methods. The traditional evaluation method of English learning classroom teaching is usually based on a series of evaluation indicators, which are scored subjectively by students, teachers themselves, peer teachers and college leaders, and then weighted. Due to the strong subjectivity and many human factors, the evaluation method is relatively narrow and limited. However, the research is just to overcome the defects of multi-subject subjective evaluation. From the perspective of analyzing students’ behaviors in English learning classroom, it provides a more intelligent method for the evaluation of students’ participation and enthusiasm in the classroom.

## Data availability statement

The original contributions presented in the study are included in the article/supplementary material, further inquiries can be directed to the corresponding author.

## Author contributions

ML completed the conception of the manuscript and writing of the first draft. DL completed the collection of materials and the collation of data. FX completed polishing, proofreading, and modification of the manuscript. All authors contributed to the article and approved the submitted version.

## Conflict of interest

The authors declare that the research was conducted in the absence of any commercial or financial relationships that could be construed as a potential conflict of interest.

## Publisher’s note

All claims expressed in this article are solely those of the authors and do not necessarily represent those of their affiliated organizations, or those of the publisher, the editors and the reviewers. Any product that may be evaluated in this article, or claim that may be made by its manufacturer, is not guaranteed or endorsed by the publisher.
